# Bipolar disorder in pregnancy and childbirth: a systematic review of outcomes

**DOI:** 10.1186/s12884-016-1127-1

**Published:** 2016-10-28

**Authors:** Marie Rusner, Marie Berg, Cecily Begley

**Affiliations:** 1 Department of Research, Södra Älvsborgs Hospital, Brämhultsvägen 53, SE-501 82 Borås, Sweden; 2 Institute of Health and Care Sciences, Sahlgrenska Academy, University of Gothenburg, Gothenburg, Sweden; 3 Centre for Person-Centred Care (GPCC), University of Gothenburg, Gothenburg, Sweden; 4 School of Nursing and Midwifery, Trinity College Dublin, Dublin 2, Ireland

**Keywords:** Bipolar disorder, Affective disorders, Mania, Pregnancy, Postpartum, Delivery, Parturition, Childbirth

## Abstract

**Background:**

Bipolar Disorder (BD) is a mental disorder usually diagnosed between 18 and 30 years of age; this coincides with the period when many women experience pregnancy and childbirth. As specific problems have been reported in pregnancy and childbirth when the mother has BD, a systematic review was carried out to summarise the outcomes of pregnancy and childbirth, in mother and child, when the mother has BD diagnosed before pregnancy.

**Methods:**

An *a priori* protocol was designed and a systematic search conducted in PubMed, CINAHL, Scopus, PsycINFO and Cochrane databases in March 2015. Studies of all designs were included if they involved women with a diagnosis of bipolar disorder prior to pregnancy, who were pregnant and/or followed up to one year postpartum. All stages of inclusion, quality assessment and data extraction were done by two people. All maternal or infant outcomes were examined, and narrative synthesis was used for most outcomes. Meta-analysis was used to achieve a combined prevalence for some outcomes and, where possible, case and control groups were combined and compared.

**Results:**

The search identified 2809 papers. After screening and quality assessement (using the EPHPP and AMSTAR tools), nine papers were included. Adverse pregnancy outcomes such as gestational hypertension and antepartum haemorrhage occur more frequently in women with BD. They also have increased rates of induction of labour and caesarean section, and have an increased risk of mood disorders in the postnatal period. Women with BD are more likely to have babies that are severely small for gestational age (<2nd-3rd percentile), and it appears that those women not being treated with mood stabilisers in pregnancy might not have an increased risk of having a baby with congenital abnormalities.

**Discussion:**

Due to heterogeneity of data, particularly the use of differing definitions of bipolar disorder, narrative synthesis was used for most outcomes, rather than a meta-analysis.

**Conclusions:**

It is evident that adverse outcomes are more common in women with BD and their babies. Large cohort studies examining fetal abnormality outcomes for women with BD who are not on mood stabilisers in pregnancy are required, as are studies on maternal-infant interaction.

**Electronic supplementary material:**

The online version of this article (doi:10.1186/s12884-016-1127-1) contains supplementary material, which is available to authorized users.

## Background

Bipolar Disorder (BD) is a severe affective mood disorder characterized by a wide range of lifelong mood changes varying between depressive, hypomanic, manic or mixed episodes [1, 2]. The lifetime prevalence for the most common variations within the BD spectrum, BD-I and BD-II, is reported as 1–2.4 % [3, 4]. A recent study from Sweden reports that incidence and prevalence of BD has increased during the last 20 years [5]. BD entails a significant burden for those that are affected as well as high societal costs related to health care, sick leave and early retirement [6]. The Swedish study has found that in persons with BD, despite similar education levels, employment levels are lower and disposable income is less compared to the general population. Also, persons with BD have more sick leave compared to the general population [5]. BD is a severe condition, and an example of this is that the suicide risk in people with this disorder is about 20–30 times greater than that for the general population [7].

The time when many women experience pregnancy, childbirth and early postpartum overlaps the period between 18 and 30 years when most women are diagnosed with BD [8]. Being pregnant and giving birth to a child is a key life transition event comprising engagement, growth and transformation [9]. Women with mental illness, however, often experience problems and increased risk of complications related to childbearing [10]. Female patients with psychiatric disorders seem to have more negative attributes with regard to sexuality and reproduction [11]. The complex picture of problems that women with BD face include: sexually risky behaviour during episodes of mania, health related risks for the mother and/or the baby, decisions regarding medication during pregnancy, as well as decisions related to unplanned/undesired pregnancy, and sexually transmitted infections [12–16].

As a basis for developing high quality healthcare with best health outcomes for mother and child when the pregnant woman has BD, there is need for a scientific overview of the risks. The aim of this paper is, through a systematic literature review, to summarise outcomes of pregnancy and childbirth, in mother and child, when the mother has bipolar disorder diagnosed before pregnancy.

## Methods

The research question guiding this systematic review was: What are the outcomes of pregnancy and childbirth (childbirth defined as labour and birth, and the first year postpartum) for women with bipolar disorder and their fetus/infant, when compared with outcomes for women with bipolar disorder, who are not pregnant, experiencing labour and birth, or postnatal. We were open to all scientific findings in the field.

### Search strategy

The use of the accepted definition of bipolar disorder [1, 2] was planned. An *a priori* protocol was designed, outlining the aim and procedure for the review and is available in the Additional file 1. An inclusion/exclusion criteria list was developed from identifying relevant PICOS (Table 1), and then tested with the PRESS criteria [17], to guide inclusion of all relevant studies. A comprehensive and systematic search was done in the following databases: PubMed, CINAHL, Scopus, PsycINFO and Cochrane, from inception until 10th of March 2015, to identify studies or existing systematic reviews. The final search string for PubMed is available in the Additional file 1, Table 2. A repeat search in PubMed and Cochrane Library was conducted in March 2016 to check for any new papers that should be included in the discussion section, for comparison.

**Table 1:** PICOS and Key words

PICOS	Key words
Population: Women with established diagnosis of bipolar disorder prior to pregnancy	Bipolar disorder OR affective disorders, psychotic OR affective psychosis OR mania
Intervention/Exposure: Pregnancy, labour and birth, and the first year post-partum	Pregnancy OR postpartum period OR delivery, obstetric OR parturition OR abortion, spontaneous OR abortion, induced OR childbirth
Comparison: Women with bipolar disorder, not experiencing pregnancy or childbirth	Women with bipolar disorder, not experiencing childbirth
Outcome: All maternal or infant health outcomes. Papers that compared effects of using different pharmaceutical treatments were excluded.	The terms ‘woman, fetus, neonate and infant’
Study design: Qualitative studies, Meta-syntheses, Surveys, Cross-sectional studies, Case reports, Experimental studies (RCTs), Quasi-experimental studies, Observational studies, Systematic reviews, Meta-analyses	Qualitative, Meta-syntheses, Surveys, Cross-sectional, Case reports, Experimental, Randomised controlled trials, Quasi-experimental, Observational, Systematic reviews, Meta-analyses

**Table 2:** Final search string for PubMed

ᅟ	ᅟ
Bipolar disorder AND (pregnancy OR postpartum period OR delivery, obstetric OR parturition OR abortion, spontaneous OR abortion, induced)	
(“bipolar disorder”[MeSH Terms] OR (“bipolar”[All Fields] AND “disorder”[All Fields]) OR “bipolar disorder”[All Fields]) AND ((“pregnancy”[MeSH Terms] OR “pregnancy”[All Fields]) OR (“postpartum period”[MeSH Terms] OR (“postpartum”[All Fields] AND “period”[All Fields]) OR “postpartum period”[All Fields]) OR (“delivery, obstetric”[MeSH Terms] OR (“delivery”[All Fields] AND “obstetric”[All Fields]) OR “obstetric delivery”[All Fields] OR (“delivery”[All Fields] AND”obstetric”[All Fields]) OR “delivery, obstetric”[All Fields]) OR (“parturition”[MeSH Terms] OR “parturition”[All Fields] OR “delivery, obstetric”[MeSH Terms] OR (“delivery”[All Fields] AND “obstetric”[All Fields]) OR “obstetric delivery”[All Fields]) OR (“abortion, spontaneous”[MeSH Terms] OR (“abortion”[All Fields] AND “spontaneous”[All Fields]) OR “spontaneous abortion”[All Fields] OR (“abortion”[All Fields] AND “spontaneous”[All Fields]) OR “abortion, spontaneous”[All Fields]) OR (“abortion, induced”[MeSH Terms] OR (“abortion”[All Fields] AND “induced”[All Fields]) OR “induced abortion”[All Fields] OR (“abortion“[All Fields] AND “induced”[All Fields]) OR “abortion, induced”[All Fields]))	

We limited our search to peer-reviewed papers. We planned to include randomised trials if the control groups could provide outcomes due to BD, but none were found.

**Fig. 1:**
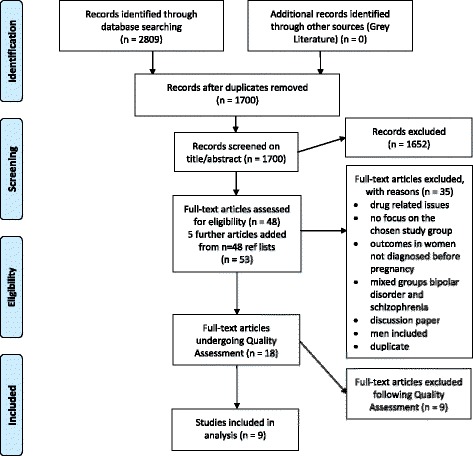
Study selection flow diagram

A total of 2809 papers were found in the five databases (PubMed *n* = 877, CINAHL *n* = 240, PsychINFO *n* = 512, Scopus *n* = 1156 and Cochrane *n* = 24). After deletion of duplicates by two reviewers (MR, MB), 1700 papers remained. Exclusion of papers by title and abstract was made by two reviewers (MR, MB) based on assessment of the inclusion and exclusion criteria. In case of disagreement the third reviewer (CB) was involved. This process ended in exclusion of 1652 papers. The full text of the remaining papers (*n* = 48) was read by three teams of two researchers each (MR/MB, MR/CB, MB/CB). Five further studies were added from their reference lists. Of these 53, 35 papers were excluded (Fig. 1). Reasons for exclusion were: drug related issues (21 papers, [14, 18–37]), no focus on the chosen study group (six papers, [38–43]), outcomes in women not diagnosed before pregnancy (two papers, [44, 45]), mixed groups bipolar disorder and schizophrenia (two papers, [46, 47]), discussion paper (two papers, [48, 49]), gender mixed groups also including men (one paper, [50]), duplicates (one paper, [15]). Occasionally it was difficult to separate the different diagnoses that were included in the sample; for example, in Doyle et al. [46] it was not possible to find separate data for the three included groups: BD I, BD II and schizoaffective disorders bipolar type. In Mac Cabe et al. [47] it was difficult to determine whether women with additional diagnoses were included in the sample or not, so both these papers were excluded. Eighteen papers (15 studies and 3 reviews) were thus quality assessed by the three pairs.

A repeat search in PubMed and Cochrane Library was conducted in March 2016, using the same methodology, to check for any new papers that should be included in the discussion section, for comparison. Fifty-eight papers were found in PubMed in this second search and no papers in the Cochrane Library. Four new papers [51–54] were found to be topical for this review. Logsdon et al. 2015 [51] described maternal-infant interaction at 12 months postpartum in women with BD compared to women with unipolar depression and a control group without a major mood disorder. Marengo et al. 2015 [52] looked at how women with BD made reproductive decisions. Both of these papers are included in the discussion section only, for noting, as they were published too late to be included in the review. Taylor et al. 2015 [53] was excluded, as their description of the characteristics of pregnant women and their use of psychotropic medication was not relevant for this review. The fourth paper, a review and meta-analysis by Wesseloo et al. 2016 [54] was also not relevant for inclusion as it included women who had a psychotic or manic episode following childbirth, even if they had never had a diagnosis of BD in pregnancy.

### Quality assessment

To assess the studies for quality the validated Effective Public Health Practice Project (EPHPP) tool [55] was used for original studies (*n* = 15), based on six categories including study design, selection bias and data collection methods. Each item was given a rating of strong, moderate or weak, based on how well the criteria were met. An overall strong, moderate or weak rating was awarded for studies with no, one, or two or more “weak” categories. We excluded six studies rated as “weak” [56–61]. The quality of the reviews (*n* = 3) was assessed using the AMSTAR tool [62, 63]. Each of the eleven issues is rated with Yes, No, Can’t answer or Not Applicable. Every yes is given one mark; 8 to 11 marks is “high quality”, 4 to 7 is “medium”, and 0 to 3 is “low quality”. We excluded all three reviews due to “low quality” [64–66].

The 9 remaining papers were included, 4 of which had a “strong” rating, and 5 a “moderate” (Table 3).

**Table 3:** Characteristics of 9 included studies

First author, year of publication	EPHPP rating	Aim of study	Study design	Diagnostic tool	Sample	Pharmaceutic treatment	Length of follow-up	Country
Akdeniz 2003 [68]	M	To evaluate and emphasise the impact of clinical and psychosocial risk factors associated with pregnancy and/or the postpartum period during the course of BD in women who had given birth	Retro-spective cohort study	BD (DSM-IV). Different types are not reported.	72 women with BD (252 pregnancies and 160 childbirths) Analysed a sub group: (*n* = 23): every mood episode that began during pregnancy and in postpartum period following birth at gestation 26 weeks+	Of women with post-partum episodes (*n* = 26) 21 had received psychotropic medication. None of the women with post-partum episodes took Lithium during pregnancy.	Pregnancy, birth and up to one month post-partum	Turkey
Bodén 2012 [69]	S	To investigate the risks of adverse pregnancy and birth outcomes for treated and untreated bipolar disorder during pregnancy.	Retro-spective case control study	BD (ICD-10 codes F30-31)	A cohort of 332,137 women 2005–2009. Women with a record of at least two BD diagnoses (*n* = 874), 320 treated with mood stabiliser, 554 untreated, compared with all other women giving birth (*n* = 331,263)	Treated BD with mood stabiliser (Lithium, antipsycotics or anticonvulsants)	Pregnancy and birth	Sweden
Di Florio 2013 [73]	M	To investigate the occurrence and timing of perinatal mood episodes in women with BD-I and BD-II	Retro-spective cohort study	BD-I and BD-II (DSM- IV)	1212 women (980 with BD-I (1404 births), 232 with BD-II (424 births)	Pharmacotherapy not reported.	Pregnancy, birth and up to one year postpartum	United Kingdom
Di Florio 2014 [74]	M	To test the hypothesis that risk of perinatal mood episodes is greater after first pregnancy	Retro-spective cohort study	BD-I and BD-II (DSM-IV)	1212 women (934 with BD-I (1404 births), 278 with BD-II (424 births)	No details on drug management reported.	Pregnancy, birth and up to one year postpartum	United Kingdom
Grof 2000 [75]	M	To examine statistically the clinical course of 28 women with typical bipolar disorder, type I, who became pregnant prior to receiving successful lithium prophylaxis	Retro-spective case/control study	BD (Re-search Diagnostic Criteria, (Spitzer et al. 1978))	28 women with BD (56 pregnancies) and no Lithium prophylaxis; 33 childless women with BD (controls)	None had prophy-lactic Lithium during pregnancy, but 4 took Lithium for depression towards end of pregnancy. Women with acute episodes received Lithium as treatment.	Pregnancy, birth and up to 9 months postpartum	A world-wide ethnic popul-ation (the majority from Canada).
Jablensky 2005 [67]	M	To determine the frequency, nature, and severity of 25 obstetric complic-ations in women with affective disorders and those with no psychiatric disorder	Case control study	BD (ICD-9 codes 296.0 and 296.2–5)	763 women with BD, 1,301 pregnancies; 1,831 women (3,129 pregnancies) with no history of mental health difficulties (controls)	No specific information on prescription of medication available.	Pregnancy and birth	Australia
Lee 2010 [70]	S	To investigate pregnancy outcomes among women with bipolar disorder, compared with women with no history of mental illness, using nationwide population-based data	Retro-spective case control study	BD (ICD-9-CM codes 295.XX, 296.0X, 296.1X, 296.4X, 296.5X, 296.6X, 296.7X, 296.80 or 296.89)	337 women with BD; 528,061 women with no history of mental health difficulties (controls)	Information on medical treatment not reported.	Pregnancy and birth	Taiwan
Mei-Dan 2015 [71]	S	To evaluate the risk of adverse perinatal outcomes among pregnant women previously hospitalised for BD	Population based case control study	BD (ICD-9, ICD-10CA, DSM-IV)	1859 women with BD; 3724 women with major depressive disorder (controls); 432,358 women with no mental illness (controls)	No information regarding medical treatment.	Pregnancy, birth and up to 27 days postpartum	Canada (Ontario)
Munk-Olsen 2009 [72]	S	To compare mothers and nonmothers to assess whether childbirth increases the risk for psych-iatric readmission and to identify pre-dictors of psychiatric readmission during the first 12 months postpartum.	Population cohort register study, prospec-tively studied	BD (ICD-8 codes 296.19, 296.39 and 298.19. ICD-10 codes F30,F31, F34.0 and F38)	All women born in Denmark between Jan 1, 1955 and July 1, 1990 who were alive on their 15th birthday and who had at least 1 psychiatric admission during the study period: January 1, 1973, through June 30, 2005. Analysis group 2 contained 56 women with bipolar disorder	No data on pharmacological treatment available.	Postpartum up to 12 months	Denmark

*BD* Bipolar Disorder, *S* Strong, *M* Moderate

### Data extraction and analysis

Three teams of two reviewers extracted key findings from each study, as well as potential explanations given by the authors for results, and their recommendations, using a pre-designed data collection form. Any disagreements were resolved by consensus. Meta-analysis was not always possible because of heterogeneity in the majority of studies (due to differing definitions of BD, or non-similar cohorts), so a descriptive narrative synthesis was used for most outcomes. When appropriate (similar definitions of BD), simple meta-analysis was used to achieve a combined prevalence. In a few cases, we were able to combine and compare case and control groups, when similar methods had also been used in two or more studies. Odds ratios were computed using MedCalc online statistical software (https://www.medcalc.org/download.php) and heterogeneity between studies was calculated using RevMan, the Cochrane Collaboration software (Review Manager, 2014). The study by Jablensky et al. [67] had a mixed cohort that included only 55 % of women with pre-existing BD, so has been left out of all meta-analyses performed.

## Results

In the following pages we describe the identified outcomes of pregnancy and childbirth in mothers, and their children, when the mother has BD diagnosed before pregnancy.

### Description of included studies

Characteristics of the nine included studies are presented in Table 3. Outcome data measured were risks or complications for the pregnancy, mother, fetus and/or baby. Seven of the nine papers had a retrospective design. Of these, three comprised data on outcomes in pregnancy and birth, one had postpartum data only (from 27 days to one year), and five comprised data on all these three episodes (Table 3). There were no restrictions for age, parity, ethnicity or other variables. We included papers written in the languages English, German, French and Swedish, and had no restrictions for age, parity, ethnicity or other variables.

The studies were published from year 2000 to 2015; one each performed in Turkey [68], Sweden [69], Australia [67], Taiwan [70], Canada [71] and Denmark [72]; two in United Kingdom [73, 74], and one study using data from an international data base of Lithium treated persons including Austria, Canada, the Czech Republic, Denmark, Germany and Sweden [75]. Four of the publications were cohort studies, and five case control studies where outcomes of pregnancy were compared for women with BD and those without. The objectives of the papers varied.

Definitions of bipolar disorder used in the papers varied from BD (DSM-IV not specified), BD-I and BD-II (DSM-IV), BD (ICD-10 codes F30-31, F34.0 and F38), RDC [76], BD (ICD-9 codes 296.0 and 296.2-5), BD (ICD-8 codes 296.19, 296.39 and 298.19), BD (ICD-9, ICD-10CA not specified) and BD (ICD-9-CM codes 295.XX, 296.0X, 296.1X, 296.4X, 296.5X, 296.6X, 296.7X, 296.80 or 296.89). (Table 3) Authors’ definitions have been used in this review.

**Table 4:** Mood episodes in pregnancy and early postpartum period for women with bipolar disorder

First author, year of publication	Key findings: reported by authors	Potential mechanisms suggested by authors	Key recommendations made by authors
Mood episodes			
Akdeniz 2003 [68]	In 13.9 % (*n* = 10), the BD illness started in the peripartum period. Twenty-three (32 %) women with BD reported at least one mood episode during pregnancy or within the first month after childbirth. Eleven mood episodes occurred in pregnancy (11 out of 252 pregnancies (4.4 %)) and which started between 2nd and 8th pregnancy months. Mean duration of episode was 5.5 weeks (SD 3.8, range 1–12). Twenty-six mood episodes occurred in the first month postnatal (26 out of 160 births (16.3 %)). Mean duration of episode was 4.5 weeks (SD 4.9, range 1–23.5). Women who had a mood episode during their 1st pregnancy, were more likely to have another episode postpartum (OR 9.6 (95 % CI, 1.002–91.964)). Women were more likely to have a postpartum mood episode after the birth of the first child. Women who had a mood episode in the 1st post-partum period were more likely to have a mood episode in the 2nd post-partum period (OR 3.6 (95 % CI, 0.257–50.330)).	Biological factors such as onset of BD at an early age, antenatal mood episode and obstetric complications appeared to influence the risk, but psychosocial factors did not.	Need rigorous prospective studies. Avoid discontinuing lithium treatment too abruptly.
Di Florio 2013 [73] Perinatal episodes across the mood disorder spectrum	Women with BD-I: 49.8 % had a mood episode in pregnancy or the post-partum period (pregnancy 8.6 %, postpartum period within 12 months of childbirth 91.4 %). More than 20 % were affected by mania or psychotic depression in the pregnancy or the post-partum period. 25 % had an episode of non-psychotic depression. Women with BD-II: 42.2 % had a mood episode in pregnancy or the post-partum period (pregnancy 18.4 %, postpartum period within 12 months of childbirth 81.6 %). Women with BD-II had a higher incidence of any perinatal mood episode compared with women with BD-I (chi-square = 10.38, d.f. = 1, *p* < 0.002 (calculated by review authors). The mood episodes were significantly more common during the first month post-partum than during pregnancy (BD-I OR 44.5 95 % CI 26.9–76.0 and BD II OR 4.7 95 % CI 2.4–9.8).	Only most severe episodes were rated, so other less severe disturbances may not have been recorded; rates of BD recurrence in pregnancy may thus be artificially low.	Prospective longitudinal studies are needed. Women with BD should be informed of the risk of peri-natal BD episode.
Di Florio 2014 [74] Mood disorders and parity	Women with BD-I: 35 % reported an episode (mania/ psychotic depression) in the first pregnancy, 20.5 % in second pregnancy and 14.6 % in subsequent pregnancies. Rates of depression were similar across all pregnancies and postpartum periods. Women with BD-II: Rates of depression: first pregnancy 46 % and second pregnancy 33 %. A significant association between parity and mood episodes within 6 weeks postpartum was found. There was no significant association between parity and mood episodes in pregnancy or later postpartum.	Women having their first baby are more anxious and stressed, due to lack of experience. Multiparous women with BD may be more aware of the possibility of postnatal episodes, and may start treatment prophylactically.	Clinical studies on the effect of parity on mood disorders should also investigate possible effect of medication reducing the risk of perinatal relapse.
Grof 2000 [75]	Five out of 28 women had a relapse during pregnancy (18 %), all in the last five weeks of pregnancy. Seven (25 %) had a postpartum relapse: 1 to 5 times, lasting 3.5 months in average, 42 % of these were manic. Postpartum episodes were significantly more than the other two periods (before and during pregnancy). Pregnancy seemed to confer a protective effect. In pregnancy, the mean rate of recurrence was 0.14, whereas mean rate in the 9 months prior to pregnancy was 0.43 (*p* < 0.05). Duration was also less (mean of 0.9 weeks during pregnancy, compared with mean of 6.1 weeks before pregnancy, *p* < 0.01). Duration postpartum was mean 12.2 weeks compared to mean 0.9 in pregnancy, *p* < 0.001.	May be due to placental hormones increasing throughout pregnancy, and abrupt cessation after birth.	Stop psychotropic medication in pregnancy, but continue to monitor women. Maybe recommence medication in the last 6 weeks of pregnancy.
Mei-Dan 2015 [71]	3.6 % (*n* = 66) of BD were hospitalised for psychiatric reasons during the index pregnancy. (this is more than for the major depressive disorder group: 1.9 % (*n* = 69)		Women with BD need more social support
Munk-Olsen 2009 [72]	Women with previous diagnoses of BD, had the highest risk of readmission 10 to 19 days postpartum (RR, 37.22; 95 % CI, 13.58–102.04) compared with mothers with BD who gave birth 6 to 11 months earlier. Cumulative incidence of admission 0–3 months postpartum was 22 %. During the first postpartum year, 26.9 % of all women with BD predating childbirth were admitted.	May be due to decrease of hormones post birth. Pregnancy, a time of emotional well-being, provides protection from BD.	Women with BD who are pregnant or considering pregnancy need careful monitoring and relevant psychoeducation.

*BD* Bipolar Disorder, *CI* Confidence Interval, *OR* Odds Ratio

### Mood episodes (Table 4)

#### Mood episodes during pregnancy and post-partum in the women with BD

Three studies reported results both on mood episodes during pregnancy or the post-partum period. Akdeniz et al. [68] showed that 32 % of the women with BD had at least one mood episode during pregnancy or within the first month after childbirth. Di Florio et al. [73] reported that 49.8 % of the women with BD-I and 42.2 % of women with BD-II had a mood episode in pregnancy or the post-partum period. Women who had a mood episode during their 1st pregnancy were, according to Akdeniz et al. [68], more likely to have another episode postpartum (OR 9.6 (95 % CI, 1.00–91.96). Also, women who had a mood episode in the 1st post-partum period were more likely to have a mood episode in the 2nd post-partum period (OR 3.6, 95 % CI, 0.25–50.33). Di Florio et al. [73] reported that women with BD-II had a higher incidence of any perinatal mood episode compared with women with BD-I (BD-II 18.4 % and BD-I 8.6 %, (chi-square = 10.38, d.f. = 1, *p* < 0.002 (calculated by review authors)) and that the mood episodes were significantly more common during the first month post-partum than during pregnancy (BD-I OR 44.5 95 % CI 26.90–76.00 and BD II OR 4.7 95 % CI 2.40–9.80). Grof et al. [75] show that episodes occurred significantly more often in the post-partum period than before and during pregnancy.

#### Mood episodes during pregnancy

Occurrence of mood episode during pregnancy was reported in four studies. In a study by Grof et al. [75] 18 % (five out of 28 women) had a relapse during pregnancy, all in the last five weeks of pregnancy. Combining the results of Akdeniz et al. [68] and Di Florio et al. [73] (as both studies used the DSM-IV definition) gives an overall prevalence for a mood episode during pregnancy of 9.3 % (104 women out of 1116).

Di Florio et al. [74] found that 35 % of the women with BD-I reported an episode (mania/psychotic depression) in the first pregnancy, 20.5 % in second pregnancy and 14.6 % in subsequent pregnancies. For women with BD-II the rates of depression in first pregnancy were 46 % and second pregnancy 33 %. Rates of depression were similar across all pregnancies and postpartum periods.

#### Mood episodes post-partum

Occurrence of mood episodes in the post-partum period was reported in four studies. In the study by Grof et al. [75], 25 % (seven out of 28) of the women had a postpartum relapse from 1 to 5 times, lasting 3.5 months on average; 42 % of these were manic. Combining the results of Akdeniz et al. [68] and Di Florio et al. [73] for mood episodes post-partum shows a prevalence of 79.2 % (812 out of 1024 births).

There was no significant association between parity and mood episodes in pregnancy or later post-partum according to Di Florio [74]. However, a significant association between parity and mood episodes within 6 weeks postpartum was found, with primiparous women being more likely to experience an episode.

#### Admission during pregnancy

Mei-Dan et al. [71] reported that 3.6 % (*n* = 66) of the women with BD were hospitalised for psychiatric reasons during pregnancy. This is more than for women with major depressive disorder, where 1.9 % (*n* = 69) were admitted during pregnancy. Risk calculations for admission during pregnancy were not reported.

#### Readmission post-partum

Munk-Olsen et al. [72] showed in a register-based cohort study (study period 1973–2005) that 26.9 % of all Danish women with BD with previous psychiatric admission(s) before birth of their first child were readmitted during the first year post-partum. That is almost twice as high compared to women with schizophrenia-like disorders (15.7 %). The highest risk of readmission was 10 to 19 days postpartum (RR, 37.22; 95 % CI, 13.58–102.04) compared with mothers with the same diagnoses who gave birth 6 to 11 months earlier. The cumulative incidence of admission 0 to 3 months postpartum was 22 %.

**Table 5:** Obstetric complications in women with bipolar disorder

First author, year of publication	Key findings: reported by authors	Potential mechanisms suggested by authors	Key recommendations made by authors
Obstetric complic-ations			
Jablensky 2005 [67]	Women with pre-existing BD had a significantly increased risk of obstetric complications (OR 1.13, 95 % CI = 1.02–1.25), whereas those who developed BD after the index birth were at no more risk than the women without mental health difficulties (OR 1.02, 95 % CI = 0.92–1.12) (Chi-square =157.56, df = 8, *p* < 0.0001).		
Antepartum hemorrhage			
Jablensky 2005 [67]	Women with BD were more likely to have antepartum haemorrhage than pregnant women with no history of mental health difficulties (adjusted OR 1.60 (95 % CI 1.11–2.32)).	Possibly due more to clustering of adverse maternal characteristics than to any one factor	Research required into environmental and genetic reproductive risks
Placenta praevia			
Jablensky 2005 [67]	Women with BD were more likely to have placenta praevia than pregnant women with no history of mental health difficulties (adjusted OR 2.13 (95 % CI 1.15–3.94)). Women with pre-existing BD had a significantly increased risk of obstetric complications (OR 1.13, 95 % CI = 1.02–1.25), whereas those who developed BD after the index birth were at no more risk than the women without mental health difficulties (OR 1.02, 95 % CI = 0.92–1.12) (Chi-square =157.56, df = 8, *p* < 0.0001).	Possibly due more to clustering of adverse maternal characteristics than to any one factor	Research required into environmental and genetic reproductive risks.
Gestational hypertension			
Lee 2010 [70]	Women with BD were more likely to have gestational hypertension (1.5 % vs. 0.5 %) than pregnant women with no history of mental health difficulties (*p* < 0.02).	None	None
Gestational diabetes			
Lee 2010 [70]	No difference in rates of gestational diabetes between women with and without BD.	None	None
Bodén 2012 [69]	No increased risk for gestational diabetes in either treated or untreated women with BD compared to women without BD.		
Induction/elective CS			
Bodén 2012 [69]	Instrumental birth: Women without BD 24.7 %; BD without treatment 33.0 %, AOR 1.49 (95 % CI 1.24 to 1.81); BD with treatment 34.1 %, AOR 1.39 (95 % CI 1.09 to 1.79) Caesarean birth: Women without BD 16.8 %; BD without treatment 23.5 %, AOR 1.45 (95 % CI 1.18 to 1.78); BD with treatment 25.6 %, AOR 1.56 (95 % CI 1.20 to 2.03) Non-spontaneous start of labour: Women without BD 20.7 %; BD without treatment 30.9 %, AOR 1.57 (95 % CI 1.30 to 1.90); BD with treatment 37.5 %, AOR 2.12 (95 % CI 1.68 to 2.67)	Mood stabilising treatment is not necessarily the sole reason for increased risk of adverse outcomes.	Important to balance risks between treating and not treating BD.
Preterm birth			
Bodén 2012 [69]	The risk of preterm birth (before 37 weeks gestation) was increased for women with BD, both for the treated and the untreated, compared with women without BD. Women without BD 4.8 %; BD without treatment 7.6 %, AOR 1.48 (95 % CI 1.08 to 2.03); BD with treatment 8.1 %, AOR 1.50 (95 % CI 1.01 to 2.24).	None	None
Lee 2010 [70]	Women with BD were more likely to have preterm births (14.2 % vs 6.9 %) than pregnant women with no history of mental health difficulties (AOR 2.08 (95 % CI 1.53–2.83).	Smoking could be a large part of the causation	
Mei-Dan 2015 [71]	Preterm birth was defined as < 37 gestational weeks. Higher prevalence for BD women 11.4 %, 212 out of 1858) and the BD group together with the ‘major depressive disorder’ group with preterm birth (11.4 %, *N* = 405) did, together, show increased prevalence above the referent group (6.2 %, *n* = 27000). Crude OR for BD: 1.93 (95 % CI 1.67–2.23. Adjusted OR for BD: 1.95 (1.68–2.26) when the control group was the referent (=1.00), adjusted for: maternal age, income quintile, hypertension, venous thromboembolic disease, gestational diabetes mellitus, gestational hypertension, preeclampsia/eclampsia. Preterm birth defined as < 32 gestational weeks. BD did have increased risk. *N* = 34, 9.1 %. Referent group *n* = 4884, 1.1 %. AOR: 1.70 (95 % CI 1.16–2.48). Preterm birth defined as < 28 gestational weeks. No significant increased risk.	None	None

*BD* Bipolar Disorder, *CI* Confidence Interval, *AOR* Adjusted Odds Ratio, *OR* Odds Ratio

### Pregnancy and childbirth related issues and complications (Table 5)

#### Non spontaneous (artificial) start of childbirth (induction or planned caesarean section)

One retrospective cohort study [69] found that women without BD had a rate of induction of labour of 20.7 %; in women with BD without treatment it was 30.9 %, Adjusted Odds Ratio (AOR) 1.57 (95 % CI 1.30 to 1.90); in women with BD with treatment it was 37.5 %, AOR 2.12 (95 % CI 1.68 to 2.67). Women without BD had a rate of caesarean birth of 16.8 %; in women with BD without treatment it was 23.5 %, AOR 1.45 (95 % CI 1.18 to 1.78); in women with BD with treatment the rate of caesarean birth was 25.6 %, AOR 1.56 (95 % CI 1.20 to 2.03) [69].

#### Gestational diabetes

One Swedish register study examined the relationship between BD and gestational diabetes. The analysis did not find any increased risk for gestational diabetes in either pharmaceutical-treated (OR 1.18 (95 % CI 0.55–2.56)) or untreated women with BD, compared with those without (OR 1.06 (95 % CI 0.56–2.02)) [69].

#### Gestational hypertension

One population-based national study in Taiwan found that women with BD were more likely to have gestational hypertension than pregnant women with no history of mental health difficulties. The odds ratio (AOR) was 2.81 when adjusted for maternal age, education level, marital status, infant gender, parity, family months income, parental age difference and parental education level (95 % CI 2.53–3.10) [70].

#### Antepartum hemorrhage

One cohort study showed that women with BD in Australia were more likely to have antepartum haemorrhage than pregnant women with no history of mental health difficulties (unadjusted OR 1.66 (95 % CI 1.15–2.39)). When adjusting for maternal age, parity, plurality, marital status, originality, and sex, the increased risk of obstetric complications remained (AOR 1.60, 95 % CI = 1.11–2.32). However, those who developed BD after the index birth were at no more risk during pregnancy and birth than the women without mental health difficulties (OR 1.02, 95 % CI = 0.92–1.12) [67].

#### Placenta praevia

One cohort study showed that women with BD were more likely to have placenta praevia than pregnant women with no history of mental health difficulties (OR 2.04 95 % CI 1.11–3.73). After adjustment for maternal age, parity, plurality, marital status aboriginality and sex the risk remained (AOR 2.13; 95 % CI 1.15–3.94) [67].

#### Preterm birth (before 37 weeks gestation)

Three studies examined this issue and found a combined prevalence of 10.68 % for preterm birth (PTB) in women with BD (328 out of 3070), compared with a rate of 6.12 % in women with no mental health problems (79,030 out of 1,291,682). This was a statistically significant difference (Z = 10.37, *p* < 0.0001, OR 1.83, 95 % CI 1.64 to 2.06).

Bodén et al. [69] found that the risk of PTB was increased for women with BD compared with women without BD (4.80 %), both for those treated (8.10 %, AOR 1.50, 95 % CI 1.01 to 2.24) and untreated (7.60 %, AOR 1.48, 95 % CI 1.08 to 2.03), when adjusted for birth order, maternal age, cohabitation, smoking, height, alcoholism and substance misuse. Lee and Lin [70] similarly showed that women with BD were more likely to have PTB (14.20 % vs 6.90 %) than women with no history of mental health difficulties (AOR 2.08, 95 % CI 1.53–2.83), when adjusted for maternal age, education level, marital status, infant gender, parity, family months income, parental age difference and parental education level. Mei-Dan et al. [71] also found a higher prevalence of PTB for women with BD (11.40 %), (AOR 1.95, 95 % CI 1.68–2.26) compared with the control group (6.20 %), when adjusted for maternal age, parity, obesity, substance abuse and medical and obstetric complications.

Early preterm occurrence was studied in the Canadian study. Preterm birth defined as < 32 gestational weeks was increased: 9.1 % vs 1.1 %. AOR: 1.70 (95 % CI 1.16–2.48). Preterm birth defined as < 28 gestational weeks show no significant increased risk: BD 0.9 % vs 0.6 % in the referent group (AOR 1.66; 95 % CI 0.92–3.02) [71].

#### Mode of birth

Mode of birth was reported in one study, the Swedish cohort. Compared to women without BD emergency caesarean section was about 50 % increased in women with BD. Adjustment was done for birth order, maternal age, cohabitation, smoking, height, alcoholism and substance misuse. For untreated women the AOR was 1.45 (95 % CI 1.18 to 1.78), and in treated women it was 1.56 (95 % CI 1.20–2.03). Similar results were found for vaginal instrumental birth (vacuum extraction or forceps): AOR in untreated BD was 1.49 (95 % CI 1.18–1.78), and treated BD women AOR 1.39 (95 % CI 1.20–2.03) [69].

**Table 6:** Fetal and neonatal complications in babies of women with bipolar disorder

First author, year of publication	Key findings: reported by authors	Potential mechanisms suggested by authors	Key recommendations made by authors
Low birthweight			
Jablensky 2005 [67]	Defined as < percentage estimated birth weight < 10th percentile. Women with BD were NOT more likely to have LBW infants (9.9 % vs.9.3 %) than pregnant women with no history of mental health difficulties. This cohort included only 55 % of women with pre-existing BD, who had a significantly increased risk of obstetric complications (OR 1.13, 95 % CI = 1.02–1.25), whereas those who developed BD after the index birth were at no more risk than the women without mental health difficulties (OR 1.02, 95 % CI = 0.92–1.12) (Chi-square =157.56, df = 8, *p* < 0.0001).	None	None
Lee 2010 [70]	Women with BD were more likely to have low birth weight infants (9.8 % vs.5.7 %) than pregnant women with no history of mental health difficulties (AOR 1.66 (95 % CI 1.16–2.38)).	Smoking could be a large part of the causation	Monitoring of fetus, early intervention if abnormalities are noted.
SGA			
Bodén 2012 [69]	No significant results for SGA were reported for women with BD (neither for the group treated with mood stabilisers nor for those not treated).	None	None
Jablensky 2005 [67]	No difference found in SGA in women with BD compared with those with no mental health difficulties.	None	None
Lee 2010 [70]	Women with BD were more likely to have SGA (22.3 % vs.15.7 %) than pregnant women with no history of mental health difficulties (AOR 1.47 (95 % CI 1.14–1.91)).	Smoking could be a large part of the causation	Monitoring of fetus, early intervention if required.
Mei-Dan 2015 [71]	Severe SGA (<3rd percentile), was not significantly elevated in BD (*n* = 84, 4.6 %; AOR 1.15 (95 % CI 1.05–1.42) compared with the referent group (*n* = 16.823, 3.9 %). SGA (<10th percentile): BD presented increased risk compared to reference group: BD: *n* = 258 of 1859 14.1 %, reference group *n* = 54 858 12.8 %. AOR 1.17 (95%CI 1.03–1.34). Adjusted for: maternal age, income quintile, hypertension, venous thromboembolic disease, gestational diabetes, gestational hypertension, pre-eclampsia/eclampsia	None	Interventions should be evaluated, to optimise health of women with BD
LGA			
Mei-Dan 2015 [71]	Severe LGA (>97th percentile) was significantly more common among women with BD (*n* = 69, 3.8 %; AOR 1.29. 95 % CI 1.08–1.54). Reference group 2.7 %, *n* = 11 712. Adjusted for: maternal age, income quintile, hypertension, venous thromboembolic disease, and gestational diabetes, gestational hypertension, pre-eclampsia/eclampsia LGA (>90th percentile) was NOT significantly more common. BD 167 of 1859 9.1 % compared to referent without mental illness *n* = 35,158 8.2 %. AOR 1.13 (0.96.1.32).	None	Interventions should be evaluated, to optimize health of women with BD
Congenital anomalies			
Bodén 2012 [69]	Congenital malformations in infants born to women: without BD: 2.0 %; with BD without treatment with mood stabilisers: 1.9 %; with BD with treatment with mood stabilisers: 0 to 3.5 %, depending on the drug used, average 3.4 % (Numbers calculated by this review team).	None	None
Jablensky 2005 [67]	No difference found in congenital abnormalities in women with BD compared with those with no mental health difficulties.	None	None
Mei-Dan 2015 [71]	BD did present increased risk for congenital anomalies. BD *n* = 90, 0.5 %. Referent group *n* = 14 963, 3.5 %. AOR: 1.48 (95 % CI 1.20–1.82) Adjusted for: maternal age, income quintile, hypertension, venous thromboembolic disease, gestational diabetes, gestational hypertension, pre-eclampsia/eclampsia	None	None
Neonatal re-admissions			
Mei-Dan 2015 [71]	Neonatal admission, < 28 days of life. BD did have increased risk. *N* = 36, 2.0 % compared to referent *n* = 3953, 0.9 %. AOR: 2.41 (95 % CI 1.76–3.31) Adjusted for: maternal age, income quintile, hypertension, venous thromboembolic disease, and gestational diabetes, gestational hypertension, pre-eclampsia/eclampsia	None	None
Fetal distress			
Jablensky 2005 [67]	No difference in fetal distress, cephalopelvic disproportion, atypical presentation, or cord anomalies, threatened preterm labor, early rupture of the membranes, prolonged labour, low 5-min Apgar scores, neonatal mortality in women with BD compared with those with no mental health difficulties.	None	None
Boden 2012 [69]	Showed no difference in low Apgar scores between women without BD and those untreated (AOR 1.56, 95 % CI = 0.85–2.86) and treated (AOR 0.88, 95 % CI = 0.33–2.34) for BD.	None	None
Stillbirth			
Jablensky 2005 [67]	No difference found in stillbirths in women with BD compared with those with no mental health difficulties.	None	None
Mei-Dan 2015 [71]	BD did not present increased risk for stillbirth. BD *n* = 11, 0.6 %. Referent group *n* = 2235, 0.5 %. AOR 1.20 (95 5 CI 0.66–2.18), adjusted for: maternal age, income quintile, hypertension, gestational diabetes, gestational hypertension, venous thromboembolic disease, preeclampsia	None	None
Infant mortality			
Mei-Dan 2015 [71]	Infant mortality < 28 days of life. BD did not have increased or reduced risk *N* = ≤ 5, referent group *n* = 1004, 0.2 %. AOR: 0.72 (0.23–2.23). Mortality <1 year of life. No difference in risk BD *n* = 7, 0.4 % referent group *n* = 1389, 0.3 %. AOR: 0.99 (95 % CI 0.44–2.22)	None	None
Neonatal morbidity			
Mei-Dan 2015 [71]	Secondary outcome; neonatal morbidity defined as RDS (respiratory distress syndrome), Seizure, sepsis, IVH (Intravenous hyperalimentation), persistent fetal circulation, and neonatal abstinence syndrome. 1. Any of these neonatal morbidities: BD had increased risk: *n* = 96, 5.4 %. Referent group (without mental illness) *n* = 8270, 1.9 %. AOR 2.99 (95 % CI 2.44–3.66) 2. RDS: BD had increased risk. *N* = 26, 1.5 %. Referent group: *n* = 4049, 1.0 %. AOR 1.64 (95 % CI 1.13–2.39) 3. Seizure: BD had increased risk. BD *n* = 10. 0.6 %, referent group *n* = 844, 0.2 %. AOR = 2.54 (95%CI 1.31–4.90). 4. Sepsis: BD had increased risk. BD *n* = 23, 1.3 %, referent group 3178, 0.7 %. AOR: 1.80 (95 % CI 1.20–2.70) 5. IVH: BD had increased risk. BD *n* = 13, 0.7 %. Referent group *n* = 1485, 0.3 %. AOR: 2.12 (95 % CI 1.22–3.67). 6. Persistent fetal circulation. BD did NOT have increased risk. BD *n* = ≤ 5, referent group *n* = 615, 0.1 %. AOR: 1.89 (95 % CI 0.78–4.58). 7. Neonatal abstinent syndrome. BD had severe increased risk. BD *n* = 70, 1.9 %. Referent group *n* = 177, 0.0 %. AOR = 52.2 (95 % CI 36.5–74.7)Adjusted for: maternal age, income quintile, hypertension, venous thromboembolic disease, gestational diabetes, gestational hypertension, pre-eclampsia/eclampsia	None	None

*BD* Bipolar Disorder, *CI* Confidence Interval, *SGA* Small for Gestational Age, *LGA* Large for Gestational Age, *AOR* Adjusted Odds Ratio, *OR* Odds Ratio

### Fetal and neonatal outcomes (Table 6)

#### Congenital anomalies

Congenital abnormalities (CA) were examined in three studies. Jablensky et al. [67] found no difference in congenital abnormalities in women with BD (*n* = 62 out of 1,301, 4.80 %) compared with those with no mental health difficulties (*n* = 152 out of 3,129, 4.90 %), but is not included in the meta-analysis.

The combined results of the other two studies [69, 71] showed that 21,632 women without BD had a baby with congenital abnormality, out of 766,750 (2.82 %), while 175 women with BD, out of 4034, had one (4.34 %). This difference is statistically significant (chi-square = 33.59, *p* < 0.0001, OR 1.56, 95 % CI 1.34 to 1.82). There was no heterogeneity shown between the two studies: Chi^2^ = 0.02, df = 1 (*P* = 0.90); I^2^ = 0 %.

Mei-Dan et al. [71] found that BD presented increased risk for congenital anomalies (*n* = 90 out of 1859, 5.00 %) compared with the referent group (*n* = 14,963 out of 432,358, 3.50 %), when adjusted for maternal age and parity (AOR 1.48, 95 % CI 1.20–1.82). Bodén et al. [69] also found the prevalence of congenital malformations was 2 % for infants born to women without BD (i.e., the normal population). For women with BD who were not treated with mood stabilisers the rate was 1.90 %, and those women with BD who were treated with mood stabilisers had rates ranging from 0 to 3.50 %, depending on the drug used. The authors did not compare these results statistically; however, if the results for women without BD and the women with no treatment for BD are combined (6528 abnormalities out of 331,817, 2.00 %) and compared with the 12 abnormalities out of 320 women with BD who were treated (3.75 %), the difference is statistically significant (Z = 2.19, *p* < 0.03, OR 1.91, 95 % CI 1.07–3.39), indicating that medications for the treatment of BD in pregnancy are associated with fetal abnormalities.

#### Small for gestational age (SGA)

Findings were mixed in the four studies examining this outcome, and definitions differed. Boden et al. [69] used ≤2nd-3rd centile (severe SGA) and Mei-Dan et al. [71] used <3rd centile (severe SGA), while Jablensky et al. [67], Lee and Lin [70] and Mei-Dan et al’s [71] secondary outcomes used <10th centile. No significant results for severe SGA were reported for women with BD in Mei-Dan et al. [71] (4.6 % versus 3.9 %), or in Bodén et al’s study [69] (3.33 % versus 2.30 %), neither for the group treated with mood stabilisers nor for those not treated. However, combining results from the two studies gives prevalence rates of 4.14 % for women with BD (113 out of 2729), compared with 3.20 % for those without (24,418 out of 762,619), a statistically significant difference (Z = 2.79, *p* < 0.006, OR 1.31, 95 % CI 1.08 to 1.58).

Results from the three studies that used the <10th centile definition differed. Jablensky et al. [67] found no difference in women with BD compared with those with no mental health difficulties, but was not included in the meta-analysis.

Women with BD in Lee and Lin’s research [70] were more likely to have SGA (22.3 % vs.15.7 %) than those with no history of mental health difficulties (OR 1.47, 95 % CI 1.14–1.91). Mei-Dan et al. [71], in testing their secondary outcome of SGA using the <10th centile definition, found that women with BD had an increased risk of SGA compared to the reference group, 13.9 % compared to 12.7 %, AOR 1.17, 95 % CI 1.03–1.34, when adjusted for maternal age and parity. When results from these last two studies are combined (15.2 % versus 14.3 %), there is no significant difference using unadjusted odds ratio (Z = 1.19, *p* = 0.24, OR 1.07, 95 % CI 0.96 to 1.21). It would thus appear that women with BD are more likely to have babies with severe SGA (<2nd-3rd centile) but not SGA (<10th centile).

#### Large for gestational age fetus (LGA)

In Mei-Dan et al.’s [71] study, severe LGA, defined as >97th centile, was significantly more common among women with BD (3.8 %), AOR 1.29, 95 % CI 1.08–1.54, compared to women with no mental difficulties (2.7 %), when adjusted for maternal age and parity. However, when LGA was defined as >90th centile, no difference was seen between the rates for women with BD (9.10 %) compared to women without mental illness (8.2 %), AOR 1.13 95 % CI 0.96–1.32.

#### Low birthweight

Two studies measured low birthweight (LBW), defined as the birth weight <10th centile [67] or <2,500 g [70]. In Jablensky et al’s [67] study women with BD were not more likely to have LBW infants (9.9 % vs.9.3 %, OR 1.06 95 % CI 0.82–1.36) than pregnant women with no history of mental health difficulties. Women with BD in Lee and Lin’s study [70] were more likely to have LBW infants (9.8 % vs.5.7 %) than pregnant women with no history of mental health difficulties (OR 1.66, 95 % CI 1.16–2.38).

#### Fetal distress/low Apgar score

Jablensky et al. [67] found no difference in fetal distress or low 5-min Apgar scores in women with BD compared to those with no mental health difficulties. Boden et al. [69] also showed no difference in low Apgar scores between women without BD and those untreated (AOR 1.56, 95 % CI = 0.85–2.86) and treated (AOR 0.88, 95 % CI = 0.33–2.34) for BD in pregnancy.

#### Stillbirth

Neither Jablensky et al. [67] nor Mei-Dan et al. [71] found any difference in the rate of stillbirths in women with BD compared with those with no mental health difficulties.

#### Neonatal readmissions

Mei-Dan et al. [71] studied neonatal admission up to 28 days of life. Babies of women with BD did have an increased risk (2.0 %) compared to those of women with no mental health difficulties (0.9 %) AOR 2.41, 95 % CI 1.76–3.31, when adjusted for maternal age and parity.

#### Infant mortality

Infant mortality defined as death before the 28th day of life was studied by Mei-Dan et al. [71]. The risk for women with BD was the same as that for women with no mental health problems (0.2 %) AOR 0.72, 95 % CI 0.23–2.23.

#### Neonatal morbidity

Neonatal morbidity, studied by Mei-Dan et al. [71] only, was defined as RDS (respiratory distress syndrome), seizure, sepsis, IVH (intravenous hyperalimentation), persistent fetal circulation, and neonatal abstinence syndrome. The risk of having any of these neonatal morbidities was higher in women with BD (5.4 %) compared with those without mental illness (1.9 %) AOR 2.99, 95 % CI 2.44–3.66. When analysed separately, babies of women with BD had higher rates for RDS, seizure, sepsis, IVH and neonatal abstinence syndrome. No difference was seen between the two groups of women in persistent fetal circulation (AOR 1.89, 95 % CI 0.78–4.58).

## Discussion

This systematic review has presented the wide-ranging health outcomes for women with bipolar disorder and their fetuses/babies. Mental health risks to women include the occurrence of mood episodes during pregnancy, rates of which varied across the studies from 9 to 18 %. Mood episodes were more common in the postpartum period [73, 75], ranging from 25 to 79 %, with differences possibly due to small sample sizes in some studies [36, 44]. It has been suggested that this may be due to placental hormones increasing throughout pregnancy, and their abrupt cessation after birth [75]. In addition, mood episodes were found to be more common in first pregnancies [73]. Similar results were found in a recent meta-analysis [54]; this review differs in methodology from ours as it included women who had a psychotic or manic episode following childbirth, even if they had never had a diagnosis of BD in pregnancy. It also included papers that compared different medication regimes, or stopping treatment at different times, which we chose to exclude as there are so many different treatments. Perhaps because of this, our postpartum relapse rate (79 %) was much higher than that quoted by Wesseloo et al. [54].

Single studies showed some incidence of increased risks in pregnancy; induction of labour occurred in women with BD, both treated and untreated, with rates of 31 and 38 % respectively, compared with 21 % in women without BD. Caesarean section rates were similarly increased. Women with BD were also more likely to have gestational hypertension [70], antepartum haemorrhage and placenta praevia [67]. Emergency caesarean section was about 50 % increased in women with BD, and similar results were found for vaginal instrumental birth [69]. There was no increased risk of gestational diabetes found [69, 70]. Pre-term birth showed a difference (10.68 % versus 6.12 %, two studies, total population 1,294,752). Lee and Lin [70] believed that smoking could be a large part of the causation, as in their (and other) studies women with BD had a higher prevalence of smoking behaviour.

Babies of women with BD have a higher prevalence of congenital abnormalities (4.34 % versus 2.82 %, two studies, total population 770,784), although the three papers examined differed in their individual findings. The difference is likely due to the smaller sample size in Jablensky’s work [67], at just over 6,000, and to the fact that this cohort included only 55 % of women with pre-existing BD. Other factors include the separation of women treated and un-treated for BD in Boden’s study [69], and the fact that Mei-Dan’s study population [71] were women previously hospitalised for bipolar disorder, so it is likely that they were on medication for their BD symptoms. There are many studies showing that mood stabilisers do cause congenital abnormalities, and that was not the focus of this review. It would appear from the three papers summarised here that women with BD who are not being treated with mood stabilisers in pregnancy might not be at the same level of increased risk of congenital abnormalities. Lee and Lin [70] recommended active monitoring of the fetus in pregnancy, and early intervention if abnormalities are noted.

A new result demonstrated by the meta-analysis in this review was that severe SGA (<2nd-3rd centile) was increased in women with BD (4.14 % versus 3.20 %, two studies, total population 765,348), in addition to previously reported increases in SGA based on the <10th centile definition [70, 71]. Again, smoking behaviour may be the main cause, as suggested by both Lee and Lin [70] and Jablensky et al. [67].

Large for gestational age babies, low birthweight, fetal distress, low Apgar score, still-birth, neonatal readmissions, infant mortality and neonatal morbidity were all examined by individual studies only, or in two studies that could not be merged for meta-analysis. Large for gestational age babies defined as >97th centile, but not as >90th centile, were more common in women with BD [71], as were low birthweight babies [70], neonatal readmissions [71] and any neonatal morbidity [71]. Fetal distress [67], low Apgar score [67, 69], still-birth [67, 71], and infant mortality [71] showed no difference.

One paper published after the systematic review was finished [51] showed that women with BD had lower scores on maternal-infant interaction at 12 months postpartum; this is an area that would benefit from further study. Marengo et al. [52], also published after the systematic review was finished, highlighted results related to reproduction decisions of women with BD. They showed that women with BD had more unplanned pregnancies (37.7 % versus 9.6 %) and fewer planned pregnancies (32.8 % versus 78.1 %) than women in the control group. They also found that women with BD more often than those without had electively interrupted at least one pregnancy (42.4 % versus 13.5 %). These findings indicate that the complex picture of problems for women with BD, mentioned in the introduction, need to be further explored.

### Review strengths and limitations

This is the first review, to our knowledge, that systematically searched the literature and summarised the risks associated with bipolar disorder for pregnant women and their babies. A key strength is the sourcing of literature from all languages. The result is a concise, extensive review that includes outcomes for both mother and baby, and spans both physical and mental health. Given the heterogeneity of data, particularly the use of differing definitions of bipolar disorder, and of some outcomes, we were unable to perform a meta-analysis for all outcomes.

## Conclusions

A clear negative impact of bipolar disorder on mothers’ and their babies’ health has been shown. Adverse outcomes such as gestational hypertension and antepartum haemorrhage occur in pregnant women with bipolar disorder. They are also prone to increased rates of induction of labour and caesarean section, and have an increased risk of mood disorders in the postnatal period. The risk of fetuses developing severe growth retardation (<2nd-3rd centile) is increased in women with BD, and increased neonatal morbidity is also found. In addition, bipolar disorder is linked to a higher incidence of congenital abnormalities (if woman are treated with mood stabilisers) and neonatal readmissions. These negative outcomes lead to longer duration of hospital stay and increase the costs of care. Active monitoring of the fetus in pregnancy, early intervention if abnormalities are noted, encouragement to decrease smoking behaviour and close support and monitoring of the mother’s mood postpartum are all likely to bring benefits in terms of improved future health and decreased monetary outlay.

As only nine papers were suitable for inclusion in this review, and as some of the studies did not control for confounding variables, or included differing populations, further research is required. In particular, large cohort studies examining fetal abnormality outcomes for women with BD who are not on mood stabilisers in pregnancy are required, as are studies on maternal-infant interaction. More qualitative studies are also recommended regarding these women's perspectives of pregnancy, childbirth and early motherhood. The compilation of these outcomes should be used to guide more informed and supportive care for women with bipolar disorder who embark on pregnancy and motherhood.

## Additional file

Additional file 1: Bipolar disorder in pregnancy and childbirth: a systematic review of outcomes. (DOC 214 kb)

## Abbreviations

BD: Bipolar disorder
