# Supplementary material for: Bipolar disorder in pregnancy and childbirth: a systematic review of outcomes
Source: BMC Pregnancy Childbirth. 2016 Oct 28;16:331. doi: 10.1186/s12884-016-1127-1 (PMC5084442; doi:10.1186/s12884-016-1127-1)
Supplement: Additional file 1: — Bipolar disorder in pregnancy and childbirth: a systematic review of outcomes. (DOC 214 kb) [file 12884_2016_1127_MOESM1_ESM.doc]

# **Bipolar disorder in pregnancy and childbirth: a systematic review of outcomes**

## **Protocol**

### Introduction

Bipolar disorder (BD) is a severe mood disorder, characterized by a variety of often life-long mood swings pending between depressive, hypomanic, manic or mixed episodes. It is well known that BD is a condition that affects all issues in life for individuals with the diagnosis. The prevalence of BD is reported to 1-2 % and the suicide risk to 6-15 %.

BD influences the relationships, both for the person with the diagnosis as well as relatives and friends. Studies on women with BD report specific problems related to pregnancy and childbirth. Sexual risk behavior during episodes of mania (Sladen et al. 2003) as well as questions about losing control of oneself, health related risks for the mother and/or the baby, medication during pregnancy, planned/unplanned pregnancy, decisions related to unplanned pregnancy are examples of the complex picture of problems. Baker (2002) has reported an increased likelihood of separation or divorce in relationships in which one person has a diagnosis of BD. Many of these problems are health related.

A large amount of research has been done on BD. Still, outcomes of pregnancy and childbirth for women with BS is a neglected area. A systematic review of the literature may significantly contribute to the knowledge base of this issue and consequently to a better health care for women with BD in fertile age.

### Research question

What are the outcomes of pregnancy and childbirth (childbirth defined as labour and birth, and the first year post partum) for women with bipolar disorder diagnosed before pregnancy and their fetus/infant?

### PICOS and Key words

| **PICOS** | **Key words** |
| --- | --- |
| ***Population:*** Women with diagnosis bipolar disorder before pregnancy | bipolar disorder OR affective disorders, psychotic OR affective psychosis OR mania |
| ***Intervention/Exposure:*** Pregnancy, labour and birth, and the first year post-partum | The period covering pregnancy, labour and birth, and the first year post-partum  pregnancy OR postpartum Period OR delivery, obstetric OR parturition OR abortion, spontaneous OR abortion, induced OR childbirth |
| ***Comparison:*** Women with bipolar disorder, not experiencing pregnancy or childbirth | Women with bipolar disorder, not experiencing childbirth |
| ***Outcome:*** All maternal or infant health outcomes | Woman, fetus, neonate and infant |
| ***Study design:*** Qualitative studies, Meta-syntheses, Surveys, Cross-sectional studies, Case reports, Experimental studies (RCTs), Quasi-experimental studies, Observational studies, Systematic reviews, Meta-analyses | See below |

### Research Aims

To summarise published research on the possible outcomes of pregnancy and childbirth for women with bipolar disorder and their fetus/infant.

### Databases and search terms to be used to identify interventions

PubMed, CINAHL, Scopus, PsycINFO and Cochrane

### Final search string

PubMed

**bipolar disorder AND (pregnancy OR postpartum period OR delivery, obstetric OR parturition OR abortion, spontaneous OR abortion, induced)**

("bipolar disorder"[MeSH Terms] OR ("bipolar"[All Fields] AND "disorder"[All Fields]) OR "bipolar disorder"[All Fields]) AND (("pregnancy"[MeSH Terms] OR "pregnancy"[All Fields]) OR ("postpartum period"[MeSH Terms] OR ("postpartum"[All Fields] AND "period"[All Fields]) OR "postpartum period"[All Fields]) OR ("delivery, obstetric"[MeSH Terms] OR ("delivery"[All Fields] AND "obstetric"[All Fields]) OR "obstetric delivery"[All Fields] OR ("delivery"[All Fields] AND "obstetric"[All Fields]) OR "delivery, obstetric"[All Fields]) OR ("parturition"[MeSH Terms] OR "parturition"[All Fields] OR "delivery, obstetric"[MeSH Terms] OR ("delivery"[All Fields] AND "obstetric"[All Fields]) OR "obstetric delivery"[All Fields]) OR ("abortion, spontaneous"[MeSH Terms] OR ("abortion"[All Fields] AND "spontaneous"[All Fields]) OR "spontaneous abortion"[All Fields] OR ("abortion"[All Fields] AND "spontaneous"[All Fields]) OR "abortion, spontaneous"[All Fields]) OR ("abortion, induced"[MeSH Terms] OR ("abortion"[All Fields] AND "induced"[All Fields]) OR "induced abortion"[All Fields] OR ("abortion"[All Fields] AND "induced"[All Fields]) OR "abortion, induced"[All Fields]))

Scopus

( ( TITLE-ABS-KEY ( pregnancy )  OR  TITLE-ABS-KEY ( postpartum  period )  OR  TITLE-ABS-KEY ( delivery, obstetric )  OR  TITLE-ABS-KEY ( parturition )  OR  TITLE-ABS-KEY ( abortion, spontaneous )  OR  TITLE-ABS-KEY ( abortion, induced ) ) )  AND  ( ( TITLE-ABS-KEY ( "bipolar disorder" )  OR  TITLE-ABS-KEY ( "affective disorders, psychotic" ) ) )  AND  ( LIMIT-TO ( DOCTYPE ,  "ar" )  OR  LIMIT-TO ( DOCTYPE ,  "re" )  OR  LIMIT-TO ( DOCTYPE ,  "ip" ) )

PsycINFO

[((bipolar disorder OR affective disorders, psychotic OR affective psychosis OR mania) AND stype.exact("Scholarly Journals")) AND ((pregnancy OR postpartum period OR delivery, obstetric OR parturition OR abortion, spontaneous OR abortion, induced) AND stype.exact("Scholarly Journals"))](http://search.proquest.com.ezproxy.ub.gu.se/myresearch/savedsearches.checkdbssearchlink:rerunsearch/827443/SavedSearches?site=psycinfo&t:ac=SavedSearches)

Cinahl


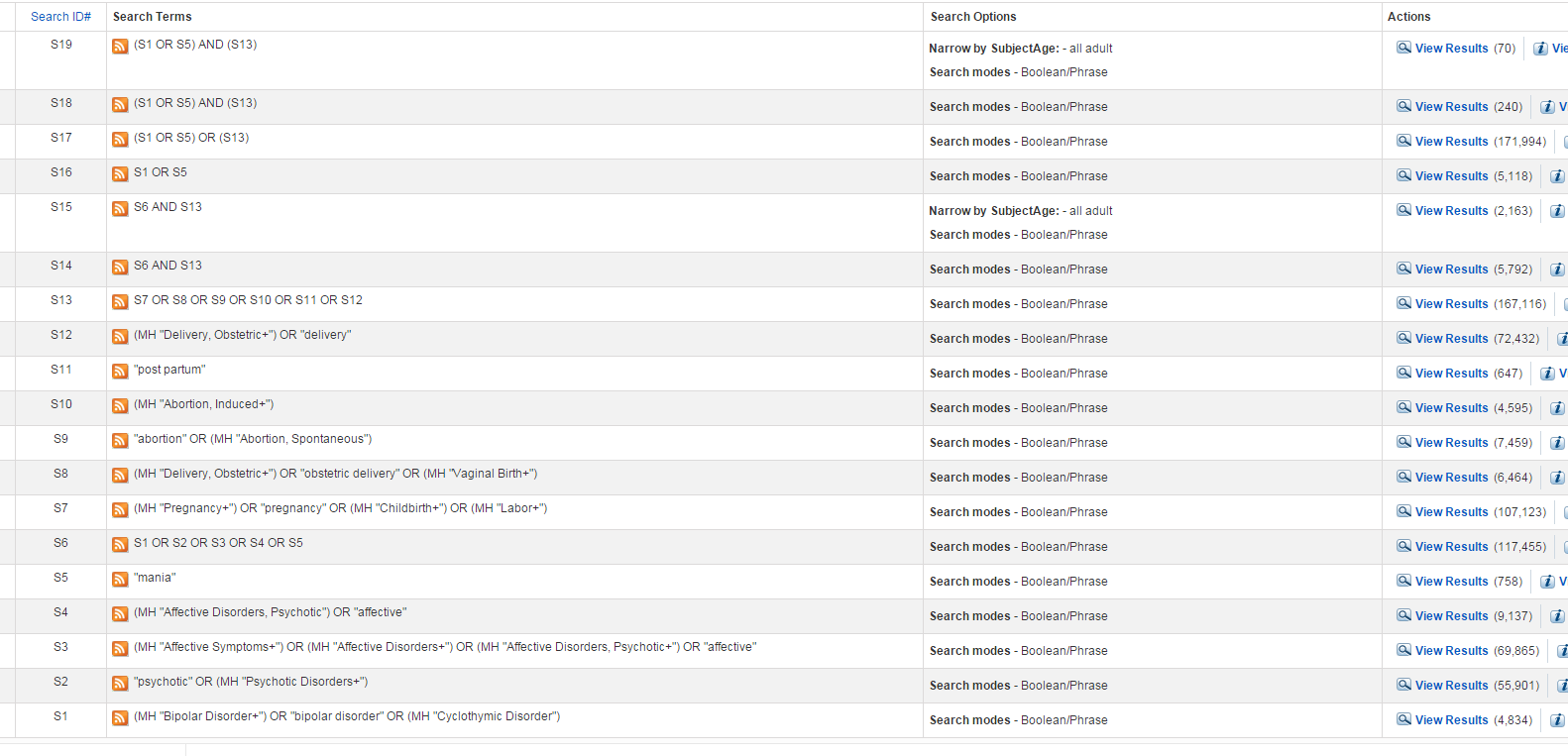


Cochrane Library

(bipolar disorder OR affective disorders, psychotic OR affective psychosis OR mania) AND (pregnancy OR postpartum period OR delivery, obstetric OR parturition OR abortion, spontaneous OR abortion, induced OR childbirth)

#### Inclusion and exclusion criteria

| **Criteria** | **Include** | **Exclude** |
| --- | --- | --- |
| Population | Women with Bipolar Disorder diagnosed before pregnancy  Any age | Men  Women without Bipolar Disorder |
| Intervention/Exposure | Pregnancy and Childbirth (childbirth defined as the period covering labour and birth, and the first year post partum) | Women who are not in a period of pregnancy or childbirth |
| Comparison | Women with bipolar disorder, not experiencing pregnancy or childbirth | Men  Women without Bipolar Disorder |
| Study design | Qualitative studies, Meta-syntheses, Surveys, Cross-sectional studies, Experimental studies (RCTs), Quasi-experimental studies, Observational studies, Systematic reviews, Meta-analyses |  |
| Outcomes | Outcomes (risks and complications) in pregnancy, labour and birth and the first year post-partum, for the pregnant woman/fetus, neonate and infant | Outcomes not related to BD (or to pregnancy, labour and birth, first year postpartum)  Papers with aim to study and compare effect on BD when using different pharmaceutical treatment (lithium, antipsychotic drugs and mood stabilizers) |
| Other | Peer-reviewed  Studies from any country  Published any time  PhD theses  Languages: English, German, French, Swedish | Reports, Position papers,  Study protocols, Reviews using just one database,  Conference proceedings, Case reports. |

## 
